# Dual Effect of Melatonin in Fetal Brain: Structural and Cellular Implications in a Rabbit Model of Intrauterine Growth Restriction

**DOI:** 10.1007/s12035-025-05032-y

**Published:** 2025-08-01

**Authors:** Laia Guardia-Escote, Yvan Gómez, Mercè Fuentes-Amell, Carlota Rovira, Eduard Gratacós, Britta Anna Kühne, Marta Barenys, Miriam Illa

**Affiliations:** 1 GRET and Toxicology Unit, Department of Pharmacology, Toxicology and Therapeutical Chemistry, Faculty of Pharmacy, University of Barcelona, Barcelona, Spain; 2 Department of Psychology, Faculty of Psychology, Universitat Rovira I Virgili, Tarragona, Spain; 3 BCNatal | Fetal Medicine Research Center, Hospital Clínic I Hospital Sant Joan de Déu, Universitat de Barcelona, Barcelona, Spain; 4 Department of Anatomical Pathology, Hospital Sant Joan de Déu, Barcelona, Spain; 5 German Centre for the Protection of Laboratory Animals (Bf3R), German Federal Institute for Risk Assessment (BfR), Berlin, Germany; 6 Institut d’Investigacions Biomèdiques August Pi I Sunyer (IDIBAPS), Barcelona and Centre for Biomedical Research On Rare Diseases (CIBERER), Barcelona, Spain; 7 Primary Care Interventions to Prevent Maternal and Child Chronic Diseases of Perinatal and Developmental Origin Network (RICORS), Instituto de Salud Carlos III, RD21/0012/0003 Madrid, Spain; 8 Sant Joan de Déu Research Institute (IRSJD), Esplugues de Llobregat, Barcelona, Spain

**Keywords:** Melatonin, Neurodevelopment, IUGR, Rabbit, Prenatal supplements

## Abstract

Intrauterine growth restriction (IUGR) is a condition in which the fetus fails to reach its genetic growth potential, often resulting in neurodevelopmental alterations. This study investigates the neuroprotective potential of gestational melatonin (MEL) treatment using an IUGR rabbit model. IUGR was induced by ligating the uteroplacental vessels of one uterine horn in pregnant rabbits, using the other horn as the control (CNT). Dams received either placebo (PLA) or MEL from gestational day 25 to 30, when pups were delivered by cesarean section. We performed a functional evaluation, followed by placental histopathology, oligodendrocyte quantification, neuronal arborization, and DAB (3, 3'-diaminobenzidine) staining of MEL receptors in the brain. IUGR groups exhibited significantly lower body weights and survival rates than CNTs, confirming successful model induction. Placental analysis indicated higher phase 2 ischemia in IUGR than CNTs, and reduced calcifications in MEL-treated groups. Brain analysis showed fewer oligodendrocytes and MEL receptors in PLA-treated IUGR groups compared to CNTs, partially reversed by MEL treatment. A dual response to MEL was observed in several endpoints, with beneficial effects on IUGR and detrimental effects on CNT groups. In conclusion, MEL demonstrates beneficial effects on the IUGR brain, particularly by increasing oligodendrocyte numbers, but its detrimental effects in CNT groups warrant caution in healthy pregnancies. These results indicate that substances can act as both therapeutic and toxic agents depending on physiological context, underscoring the need for further research to understand MEL’s mechanisms and optimize treatment strategies.

**Supplementary Information:**

The online version contains supplementary material available at 10.1007/s12035-025-05032-y.

## Introduction

Intrauterine growth restriction (IUGR) is characterized by the inability of the fetus to reach its genetic growth potential during the prenatal period, making it one of the main causes of perinatal and neonatal morbidity and mortality [1]. Evidence in humans indicates that children who experienced IUGR may have a higher risk of presenting various conditions, including both short- and long-term neurodevelopmental alterations [2, 3]. IUGR constitutes a challenging public health problem, as postnatal treatment approaches have proven insufficient to address these alterations and improve prognosis [3]. For this reason, new strategies are being investigated, including interventions during gestation. In vitro research using the neurosphere model suggested that therapies administered during gestation, such as melatonin (MEL) or docosahexaenoic acid (DHA), may have neuroprotective effect and support normal development [4]. MEL is a hormone mainly synthetized in the pineal gland and involved in many physiological and biological processes. It is primarily known for its role in regulating the circadian rhythm in vertebrates. Of special interest, MEL and its metabolites exhibit strong antioxidant properties, effectively scavenging reactive oxygen species [5, 6].

Recent studies have identified MEL as an essential hormone during pregnancy, specifically in the placenta. MEL’s antioxidant properties are necessary to ensure the homeostasis of the mother and fetus [6]. It is important to note that there is no endogenous fetal production of MEL; thus, the levels in the fetus correspond to the day/night circulating concentration of the mother. MEL crosses the placenta unchanged and sends a temporal circadian signal to the fetus, preparing the central nervous system to properly manage day/night fluctuations in the environment after birth [7]. Maternal plasma MEL levels consistently rise, reaching their peak at third trimester and decrease after delivery, indicating the existence of local endogenous production of MEL in the placenta [8]. The circadian rhythm is crucial for the internal rhythms of the fetus, and its disruption may lead to detrimental outcomes and promote chronic illness later in life [6]. Indeed, MEL has demonstrated neuroprotective properties in response to postnatal insults, enhancing myelination repair in cases of hypoxia, ischemia, inflammation and drug-induced brain injury [9, 10]. MEL is also known to regulate neuronal generation and synaptic plasticity in the dentate gyrus of adult rodents, a region particularly sensitive to inflammatory and oxidative damage [11, 12]. Indeed, Miller et al. (2014) [13] demonstrated that the non-treated brain lesions underlying neurodevelopmental delay in IUGR lambs are mainly composed of white matter hypomyelination and axonal lesions, along with notable disorganization of the cerebral cortex and selective vulnerability of the hippocampal region CA3. In contrast, prenatal MEL treatment reduces fetoplacental oxidative stress, effectively reduces cerebral white matter and gray matter lesions resulting from placental insufficiency and fetal growth restriction, and, at least at the short-term, improving neurodevelopment [13]. However, despite extensive evidence of MEL’s neuroprotective effects, concerns remain about its safety, with some studies reporting potential negative effects associated with its use [14].

The present investigation aimed to assess the neuroprotective effects of gestational MEL in a rabbit model of IUGR. Rabbits were chosen for their similarity to humans in terms of brain development, complexity, and gestational endpoints, making them a reliable model for translating data to clinical settings [15]. Additionally, the rabbit’s precocial score at birth (0.537) closely resembles that of humans (0.654) [16]. In the same line, the IUGR model was induced on gestational day (GD) 25, which corresponds to gestational weeks 28–32 in humans, a period when most cases of IUGR appear. During this neurodevelopmental stage, immature oligodendrocytes appear in the white matter and increase rapidly over time until myelination begins after birth [16–18]. Previous studies with this model have reported neurodevelopmental alterations in IUGR compared to CNT groups [19]. Based on this, we hypothesize that MEL administration during gestation will mitigate some of these neurodevelopmental deficits, highlighting its neuroprotective properties. This study seeks to build on existing evidence and explore MEL's potential as a therapeutic option to treat IUGR, with promising implications for its future application in clinical practice.

## Materials and Methods

### Animals and Care

**Image 1:**
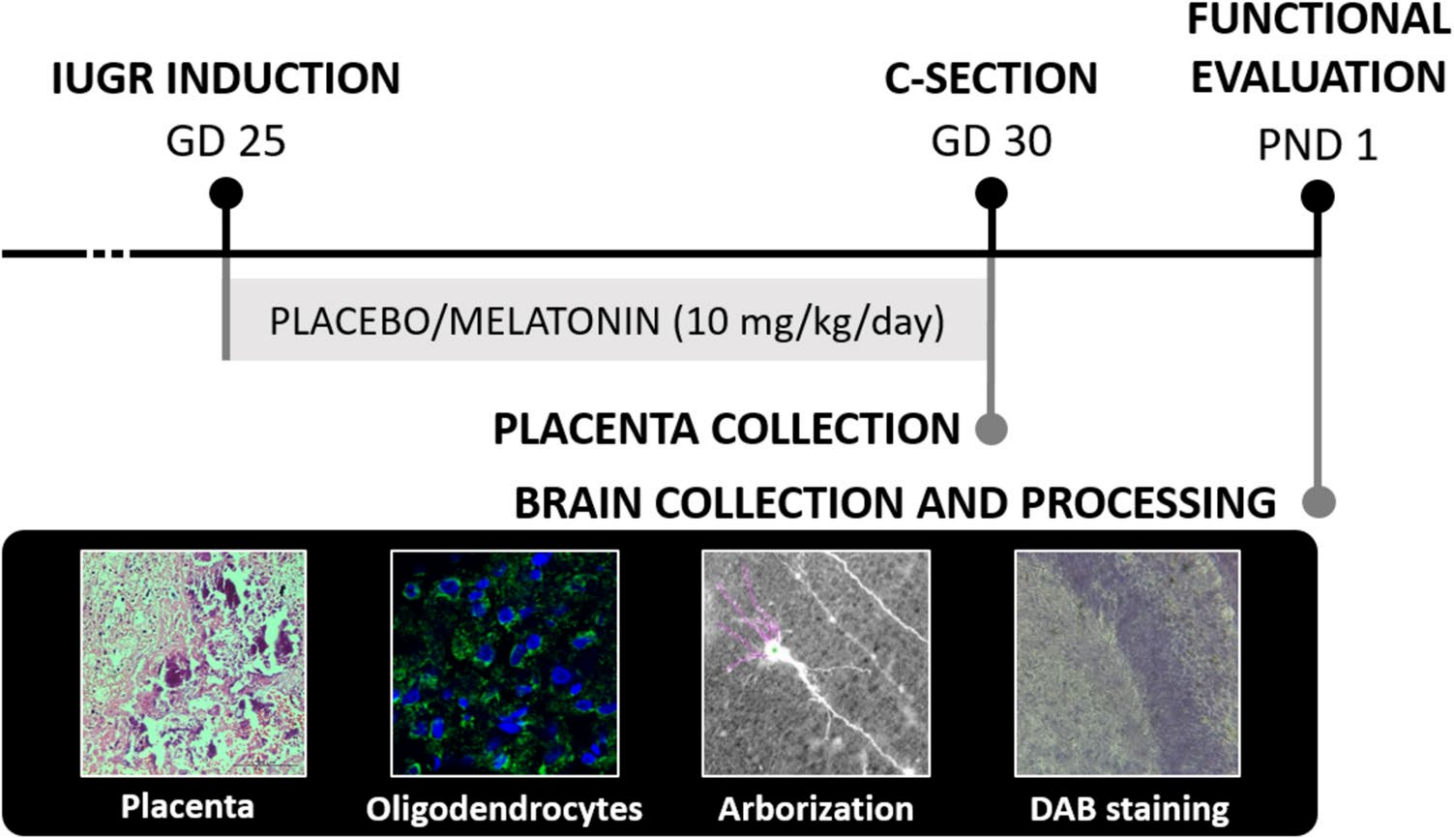
Experimental design. A total of 18 pregnant New Zealand rabbits were included in the study. On gestational day (GD) 25, rabbits underwent intrauterine growth restriction (IUGR) induction surgery. Prior to the surgery, they were randomly assigned to one of the two treatment groups: placebo (PLA) or melatonin (MEL) at a dose of 10 mg/kg/day, administered from GD 25 to GD 30. At the end of the treatment period, pups were delivered by cesarean section. On postnatal day (PND) 1, the pups underwent functional evaluation. Following the assessment, they were euthanized for sample collection. Samples were then processed for placental histopathology, O4-immunoreactive oligodendrocyte quantification, neuronal arborization analysis, and DAB staining

In the present investigation, we used 18 pregnant New Zealand rabbits, provided by a certified commercial farm (Granja San Bernardo, Spain). After the arrival at the animal facilities, pregnant rabbits were maintained in individual cages and allowed to habituate for a week before starting the treatment. All animals had access to fresh water and normal chow diet, and they were kept under standard conditions with a 12 h light/dark automatic light cycle (light on 8:00–20:00). Experimental timeline is displayed in the Image 1. The use of animals and the experimental protocol were approved by the Animal Experimental Ethics Committee (CEEA) of the University of Barcelona with the license number 03/17 and were conducted in accordance with the Spanish Royal Decree 53/2013 on the protection of experimental animals, and the European Communities Council Directive (2010/63/EU). Animal work was conducted fulfilling ARRIVE’s guidelines and is reported accordingly [20].

### IUGR Induction and Therapy Administration

At 25 days of gestation, the animals were subjected to the IUGR induction surgery. IUGR was induced by surgically ligation of the 40–50% of the uteroplacental vessels of each gestational sac (IUGR model), while the un-ligated individuals corresponded to the control (CNT) group. One uterine horn in each animal was assigned to the CNT condition, while the other was assigned to the IUGR condition. All surgical procedures were performed under general anesthesia (ketamine and xylazine), and the animals were monitored at all times to ensure appropriate depth of anesthesia.

Before the IUGR induction, each pregnant rabbit was randomly distributed in two treatment groups: ones received MEL from GD 25 to 30, and the others received placebo (PLA), for the same period. MEL or PLA were orally administered to the pregnant rabbits once per day at the same time (8:30 h a.m.) in the morning using a syringe to release the preparation directly into the mother’s mouth. The specific dose of MEL (10 mg/kg/day) was calculated taking into account previous evidence [4]. MEL solution was prepared by the Pharmacy Services of the Hospital Sant Joan de Déu (Barcelona, Spain) as an oral suspension at 20 mg/ml. The dose was calculated in a daily base, taking into account the body weight of the animal. PLA (physiological saline 0.9%) was administered in a similar volume than MEL.

At GD 30, living and stillborn pups were obtained by a cesarean section and all of them were included in the survival ratio calculations. For further analysis, an inclusion criterion was applied to the CNT group, including only pups with a body weigh higher than the 25 th percentile (39.7 g, Barenys et al. (2021) [21]) to prevent the inclusion of spontaneous IUGR in this group. For the IUGR group, only animals with a BW below 60 g were included [19]; however, all pups in the study met this criterion.

### Functional Evaluation

Functional evaluation was performed to all the alive pups on PND 1, following previously described methodology [15]. For each animal, the included variables were scored on a scale 0–3 or 0–4 by two blinded experimenters (LG-E and YG). The first part of the evaluation lasted 1 min and included: a) posture of the animal, b) tone, c) circular motion, d) locomotion movement of the head, forelimbs and hindlimbs, e) intensity of the movements, f) duration of the activity, g) lineal movement, and h) shortest fore-hindpaw distance. After that, the righting reflex (10 tries) was assessed in the same animals, followed by a) suckling and swallowing, b) head turning during feeding, and c) olfaction. The score grading is detailed in Supplementary Table 1.

### Sample Collection

Placentas were obtained after the cesarean section and washed with saline solution. After that, placentas were fixed for 24 h in formalin 10% (Sigma-Aldrich Co. LLC., Spain), stored in ethanol 30%, and finally embedded in paraffin. After functional evaluation, pups were weighed and deeply anesthetized with ketamine (35 mg/kg) and xylazine (5 mg/kg) before being euthanized by decapitation. Whole brains were collected and processed according to the corresponding analysis: neuronal arborization or oligodendrocyte analysis. Four brains from each group were selected for the Golgi-cox staining protocol and processed according to the manufacturer’s protocol (detailed below). The other brains were fixed in formalin 10% for 24 h, dehydrated with sucrose 30% for 48 h and snap-frozen by pre-chilled 2-methylbutene on dry ice. Samples were stored at −80 ºC until further use.

### Histological Procedures

#### Placenta

Five placentas randomly selected from each experimental group were included in the analysis based on previous experience working with this animal model [19, 22]. Two consecutive slices of 4 µm were obtained for each paraffin-embedded placenta using a Leica RM2235 manual rotary microtome (Leica Microsystems GmbH, Germany) and stained with hematoxylin and eosin (standard protocol). Samples were observed with a Leica DCM1000 microscope (Leica Microsystems GmbH, Germany) by two independent investigators blinded to the experimental groups (LG-E and YG) and supervised by a pathologist (CR). The agreement between the investigators and the pathologist scores was strong, indicating reliable and consistent observations. Two different regions were included: a) the decidua basalis or maternal part, and b) the labyrinth zone or fetal part. We assessed ischemia phase 1 (%), ischemia phase 2 (%), the presence of polymorphonuclear cells (Y/N), fibrosis (%), fibrin (%) and calcifications (%) for each region. Besides, for the labyrinth zone, we also studied the vascular collapse, with a score system from 0 to 5: 0) Unremarkable; 1) Minimal; 2) Mild; 3) Moderate; 4) Marked; and 5) Severe. The detailed histopathological assessment of tissue is summarized in Supplementary Table 2, outlining the key endpoints and their corresponding scoring criteria and descriptions.

#### O4-Immunoreactive Oligodendrocytes

Five brains per group were randomly selected for the O4-immunoreactive oligodendrocyte evaluation, based on previous evidence [19, 21]. Two coronal sections of 40 µm were obtained for each brain with a Leica DCM1000 cryostat (Leica Microsystems GmbH, Germany), and three different regions in the corpus callosum were analyzed within each section. The corpus callosum was selected because previous MRI studies have identified alterations in white matter and a reduced number of pre-oligodendrocytes in IUGR compared to CNT [19, 23]. The sections were processed as previously described [15]. Briefly, after two washing steps with PBS and one permeabilization step with PBST 0.3%, samples were incubated with blocking solution for 1 h at room temperature (RT). After that, the slices were incubated with the primary antibody Mouse IgM anti-O4 (Sigma-Aldrich) overnight at 4º C. The next day, after three washing steps with PBST 0.3%, slices were incubated for 1 h with the secondary antibody goat anti-mouse IgM (Invitrogen) at RT with 1% Hoechst nucleic acid stain (Fisher Scientific). After one washing step with PBST 0.3%, two washing steps with PBS, and one with distilled water, slices were mounted with Fluoromount-G™ (Invitrogen, USA) and then sealed. After immunostaining, 40X fluorescent images were taken on a Leica TCS SP8 microscope (Leica Microsystems GmbH, Germany) with 10 steps in 1 µm in the Z-stack. ImageJ software was used to count the number of cell nuclei and the number of cells stained with positive O4 fluorescence around the nucleus.

#### Neuronal Arborization (Golgi-Cox Staining)

Four brains per group were randomly selected for the study of neuronal arborization, based on previous evidence [19, 24]. Brains were processed using a FD Rapid GolgiStain™ Kit (FD Neurotechnologies Inc., USA), according to the manufacturer’s protocol. A Leica CM3050S cryostat (Leica Microsystems GmbH, Germany) was used to obtain 100 µm sections from each brain. Three pyramidal neurons were selected from layers II and III of the frontal cortex from each brain hemisphere (6 neurons per slice), and one image per neuron was obtained. Images were taken on a Leica TCS SP8 microscope (Leica Microsystems GmbH, Germany). Several parameters for each neuron were obtained using ImageJ software, with the Neuroanatomy package plugin SNT. The parameters included: a) the area of the soma, b) the number and length of basal dendrites c) the number and length of secondary dendrites, and d) the basal dendritic complexity, by using the Sholl technique.

### DAB (3, 3’-Diaminobenzidine) Staining

MEL receptor 2 (MT2) was studied in four brains randomly selected per group by DAB staining. Brain sections were obtained with a Leica DCM1000 cryostat (Leica Microsystems GmbH, Germany) and processed according to the manufacturer’s protocol. Briefly, the samples were washed with TBS plus 0.025% Triton X-100, blocked with 10% rabbit serum with 1% BSA in TBS for 2 h and incubated with H_2_O_2_ in TBS for 15 min. After washing the sections with TBS plus 0.025% triton X-100, they were incubated with the primary antibody anti-MT2 A (Sigma-Aldrich) overnight. After a washing step, they were incubated with HRP polymer secondary antibody. The samples were incubated with the DAB substrate (DAB substrate kit, abcam, UK) for 5 min, washed and counterstained using hematoxylin. After that, slices were mounted with Fluoromount-G™ (Invitrogen, Thermo Fisher Scientific, Inc., USA), and sealed. The samples were observed with a Leica DCM1000 microscope (Leica Microsystems GmbH, Germany) and analyzed using ImageJ software.

### Statistical Analysis

Data analysis was conducted using the Statistical Package for the Social Sciences (SPSS) version 27.0 (IBM Corp, Chicago, USA) software. GraphPad Prism 10 (GraphPad Software, USA) was used for graphical representation. Data were analyzed with the litter as the experimental unit. The descriptive of the functional evaluation variables was expressed as mean and standard deviation (SD) for normal distributions, whereas median and interquartile range (IQR) were used for non-normal distributions and ordinal variables. The variance homogeneity was assessed by a Levene test. A two-way analysis of variance (ANOVA) was used to study the general effects of the model (IUGR induction) and treatment (MEL) on the different endpoints. A one-way ANOVA followed by Tukey's post-hoc test of variance was used to analyze group differences. Non-parametric data were analyzed using the Kruskal–Wallis or Mann–Whitney U test as appropriate. Statistical significance was set at *p* < 0.05 for the whole study.

## Results

### Perinatal Data

**Fig. 1:**
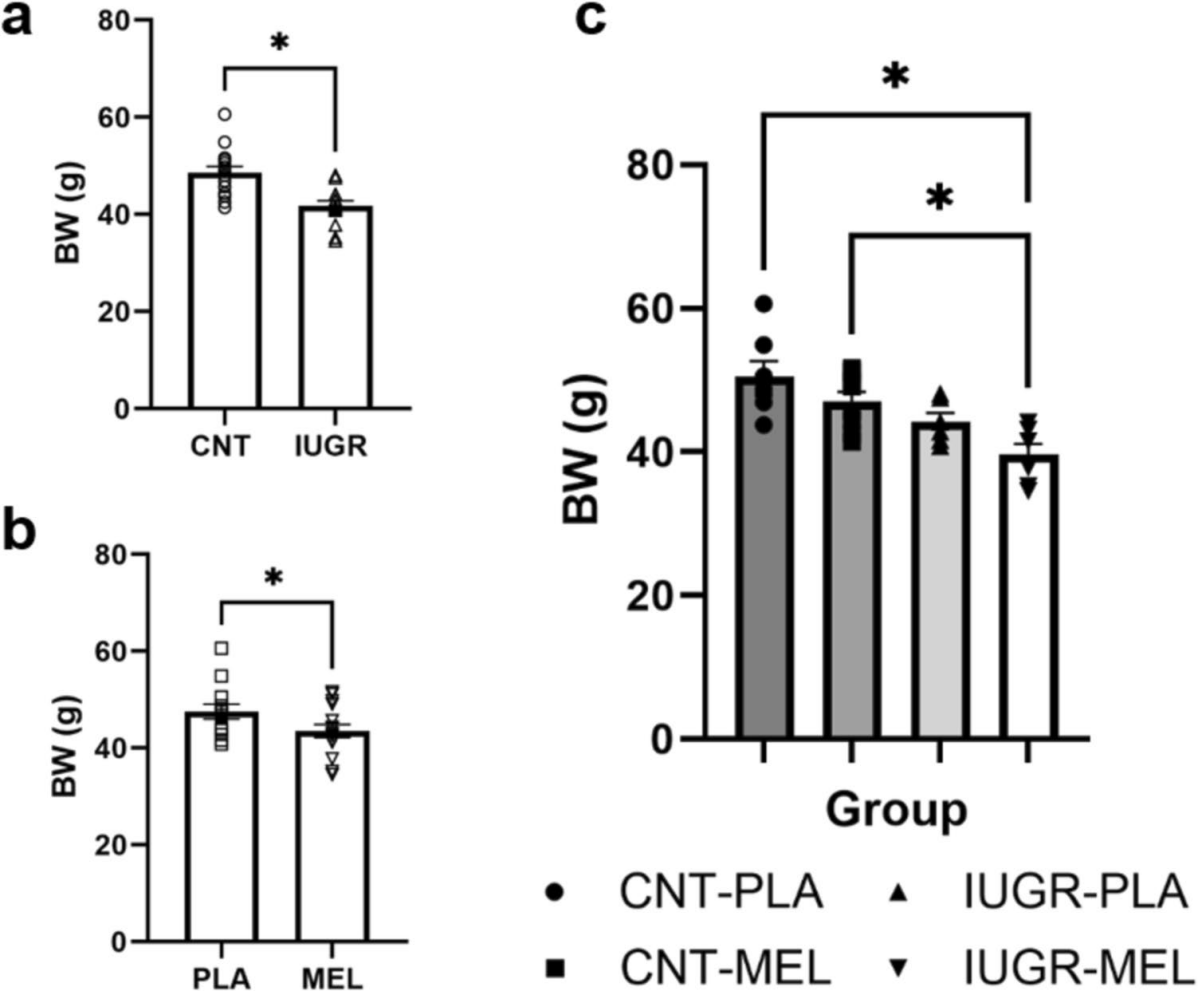
Perinatal data. General effects of the model (**a**) and the gestational treatment (**b**) on litter BW. BW according to treatment group (**c**). An asterisk (*) indicates a significant difference between groups at *p* < 0.05. Results are represented as mean ± S.E.M. Abbreviations: CNT, control; IUGR, Intrauterine growth restriction; PLA, placebo; MEL, melatonin; BW, body weight

Eighteen adult female rabbits were included in the experiment, two of which died from complications during surgery, and one had to be excluded due to a toenail infection. Consequently, fifteen mothers were divided into two treatment groups: placebo (PLA, *n* = 7) and melatonin (MEL, *n* = 8). Maternal body weight (BW) did not differ significantly between treatments (PLA: 5.19 ± 0.22 kg; MEL: 4.99 ± 0.13 kg). Offspring BW was assessed at postnatal day (PND) 0, including only the alive pups. The exact number of pups per litter is disclosed in Supplementary Table 3. A two-way ANOVA (model x treatment) revealed a significant effect of both model [F(1,24) = 17.894, *p* < 0.001] and treatment [F(1,24) = 6.254, *p* = 0.020]. As expected, the IUGR groups presented lower BW compared to the CNT groups (Fig. 1a). Additionally, the MEL treatment also decreased BW compared to PLA groups (Fig. 1b). Subsequent analysis using a one-way ANOVA (group) followed by *post-hoc* tests showed significant differences between groups [F(3,24) = 8.185, *p* < 0.001]. Specifically, the CNT groups presented higher BW compared to the MEL-treated IUGR group (*p* < 0.001 and *p* = 0.014, respectively), but CNT-MEL or IUGR-MEL did not present a significantly lower weight than their non-treated corresponding CNT-PLA and IUGR-PLA groups (Fig. 1c).

Brain weight at PND1 and the ratio between brain weight and BW were also investigated, with no differences found between groups (Supplementary Fig. S1).

**Fig. 2:**
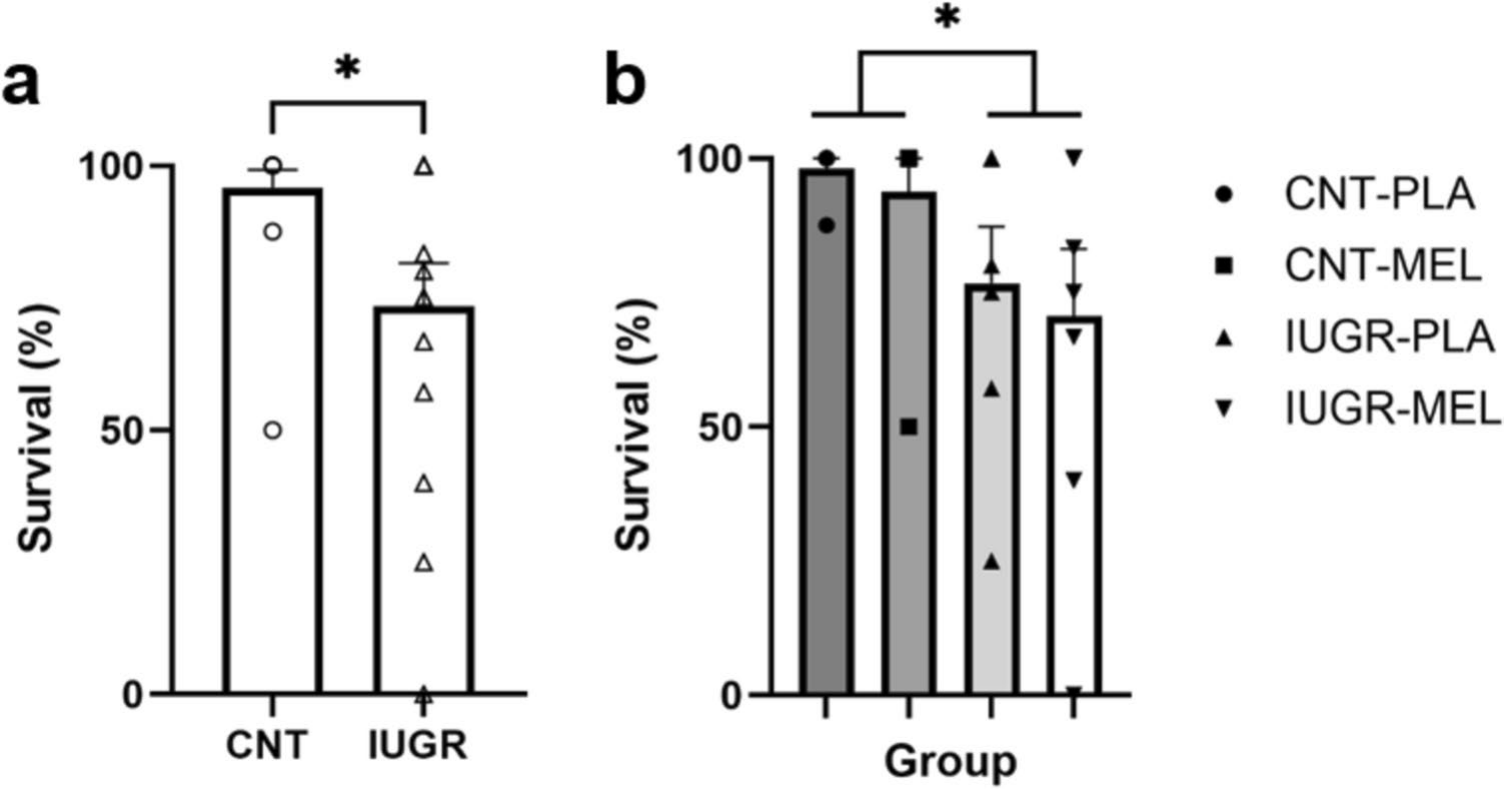
Percentage of survival. Percentage of survival based on model (**a**) and according to the treatment group (**b**). An asterisk (*) indicates a significant difference between groups at *p* < 0.05. Results are represented as mean ± S.E.M. Abbreviations: CNT, control; IUGR, Intrauterine growth restriction; PLA, placebo; MEL, melatonin

Percentage of survival was analyzed by means of a two-way ANOVA (model x treatment). Significant differences were found based on model [F(1,26) = 6.139, *p* = 0.020], indicating decreased survival in the IUGR groups compared to the CNTs (Fig. 2a-b), as expected according to the literature [19].

### Functional Evaluation

No differences were observed between the groups for any of the studied variables (Table 1).

**Table 1:** Functional evaluation

Variable	CNT-PLA n (litter) = 6	CNT-MEL n (litter) = 7	IUGR-PLA n (litter) = 6	IUGR-MEL n (litter) = 7	*p* value
Posture, score#	3.00 (1.3)	3.00 (1.00)	2.75 (0.6)	2.50 (1.0)	n.s
Righting reflex, number of turns	6.64 ± 1.90	8.48 ± 1.41	7.17 ± 1.51	8.43 ± 1.09	n.s
Tone, score#	2.75 (1.1)	3.00 (1.0)	3.00 (0.9)	3.00 (1.0)	n.s
Circular motion, score#	2.00 (2.0)	2.00 (1.0)	1.75 (1.3)	1.50 (1.0)	n.s
Head locomotion, score#	1.00 (0.8)	1.00 (1.0)	1.00 (0.9)	1.00 (0.0)	n.s
Forelimbs locomotion, score#	2.00 (1.0)	2.00 (1.0)	2.00 (0.5)	2.00 (0.0)	n.s
Hindlimbs locomotion, score#	1.50 (1.4)	2.00 (0.5)	1.50 (1.5)	1.50 (1.0)	n.s
Intensity, score#	1.75 (1.3)	2.00 (1.5)	2.00 (1.3)	2.00 (1.0)	n.s
Duration, score#	3.00 (1.5)	3.00 (0.0)	3.00 (0.5)	3.00 (0.0)	n.s
Lineal movement	3.04 ± 0.94	3.64 ± 0.90	2.94 ± 0.69	3.99 ± 1.93	n.s
Fore-hind paw distance, mm	2.72 ± 0.69	2.52 ± 1.10	3.38 ± 0.24	2.78 ± 0.41	n.s
Sucking and swallowing, score#	1.75 (2.3)	2.50 (2.0)	2.75 (1.6)	3.00 (0.5)	n.s
Head turning, score#	1.25 (1.5)	2.00 (1.5)	2.00 (0.9)	2.00 (0.5)	n.s
Olfaction, score#	2.25 (2.1)	3.00 (2.0)	3.00 (1.0)	3.00 (1.0)	n.s
Olfaction time, s	5.11 ± 0.067	4.12 ± 0.83	5.53 ± 0.15	4.98 ± 0.63	n.s

Results are expressed as median and IQR (#), except the variables “righting reflex, lineal movement, fore-hind paw distance and olfaction time” which are expressed as mean ± SD. Abbreviations: CNT, control; IUGR, Intrauterine growth restriction; PLA, placebo; MEL, melatonin; SD, standard deviation; IQR, interquartile range

### Placenta Histopathology

**Fig. 3:**
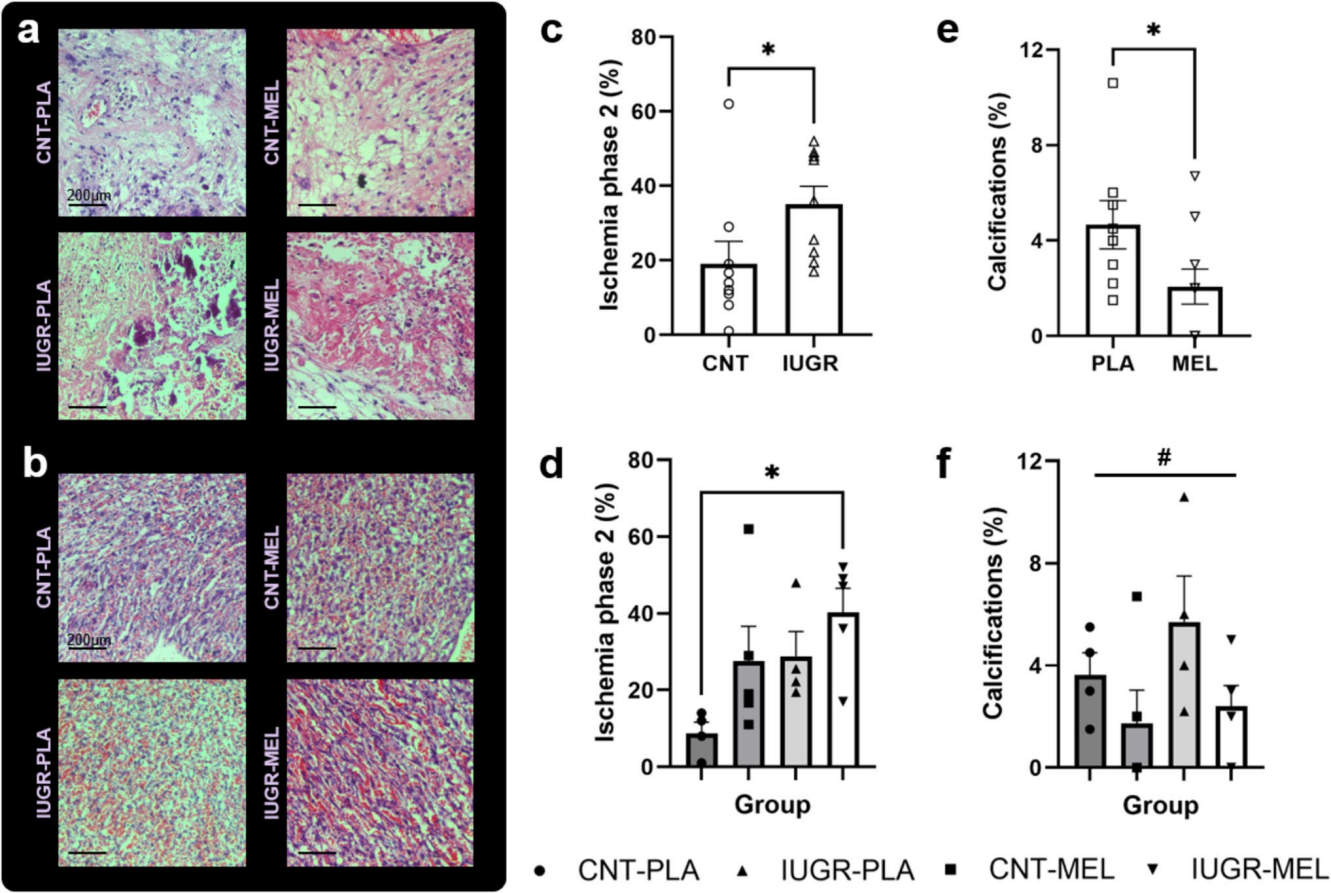
Placenta histopathology. Representative images of the histopathology in the decidua (**a**) and labyrinth (**b**) parts of the placenta. Percentage of ischemia phase 2 depending on the model (**c**) and the treatment group (**d**) on the decidua. Percentage of calcifications in the decidua based on MEL treatment (**e**), and the treatment group (**f**). An asterisk (*) indicates a significant difference between groups, while the symbol (#) represents a significant effect of the treatment at *p* < 0.05. Results are represented as mean ± S.E.M. Abbreviations: CNT, control; IUGR, Intrauterine growth restriction; PLA, placebo; MEL, melatonin

Histological differences were evaluated in both the labyrinth and decidua parts of the placenta, with differences primarily detected in the decidua section (Fig. 3a-b). Using an independent-samples Kruskal–Wallis test, significant differences were identified in the percentage of ischemia phase 2 (H = 9.030, *p* = 0.029) among groups. Further analysis found a significant influence of the model (U = 67.000, *p* = 0.019), with IUGR group presenting higher percentage of ischemia phase 2 than CNT group (Fig. 3c-d). Conversely, an independent-samples Mann–Whitney U test revealed a significant impact of the treatment on the percentage of calcifications (U = 17.500, *p* = 0.043), with MEL treatment improving this endpoint in all groups (Fig. 3e-f). No significant differences were observed in the other endpoints included in the analysis (Supplementary Fig. S2).

### O4-Immunoreactive Oligodendrocyte Results

**Fig. 4:**
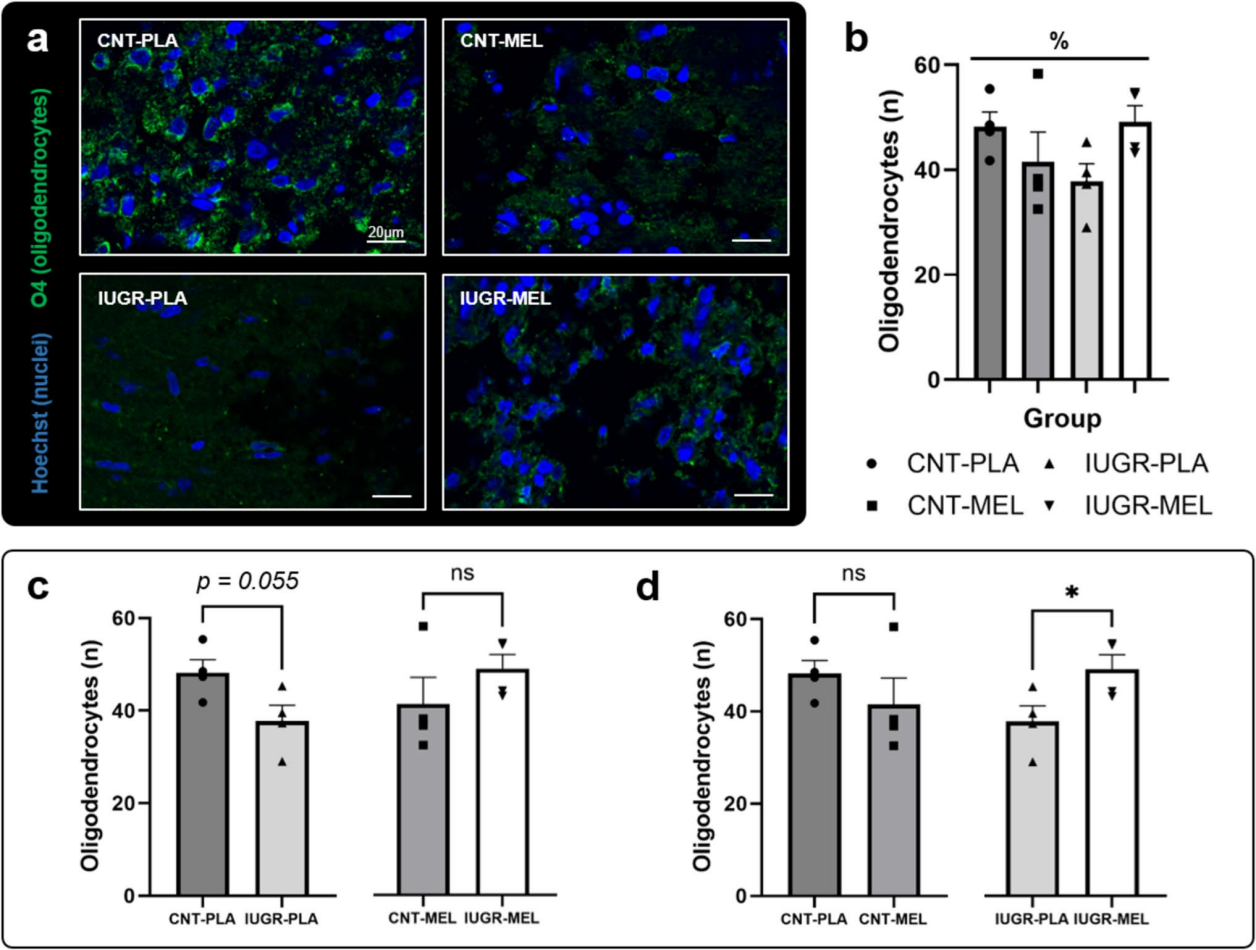
Oligodendrocyte study. Representative images of O4 + positive cells (green) and nuclei marker (Hoechst, blue), scale bar = 20 µm (**a**). Number of oligodendrocytes based on the treatment group (**b**), and pairwise comparisons depending on the model (**c**) and the treatment (**d**). An asterisk (*) indicates a significant difference between groups, while the symbol (%) represents a significant interaction between the model and the treatment at *p* < 0.05. Results are represented as mean ± S.E.M. Abbreviations: CNT, control; IUGR, Intrauterine growth restriction; PLA, placebo; MEL, melatonin

Differences in the number of oligodendrocytes were evaluated through a two-way ANOVA (model x treatment) (Fig. 4a). The analysis revealed a significant interaction between model and treatment [F(1,12) = 5.293, *p* = 0.040], indicating a dual response to MEL depending on the model (Fig. 4b). Subsequent pairwise comparisons using t-tests showed a trend in the number of oligodendrocytes between the CNT and IUGR groups treated with PLA [t = 2.380, d.f.6, *p* = 0.055], suggesting a decrease in the number of oligodendrocytes in the IUGR group compared to CNT (Fig. 4c). Interestingly, the IUGR group treated with MEL presented an increase in the oligodendrocyte number compared to the PLA-treated IUGR group [t = 2.472, d.f.6, *p* = 0.048], thus reversing the previously observed detrimental effect (Fig. 4d). No significant differences were observed in the other parameters included in the analysis (Supplementary Fig. S3).

### Neuronal Arborization

**Fig. 5:**
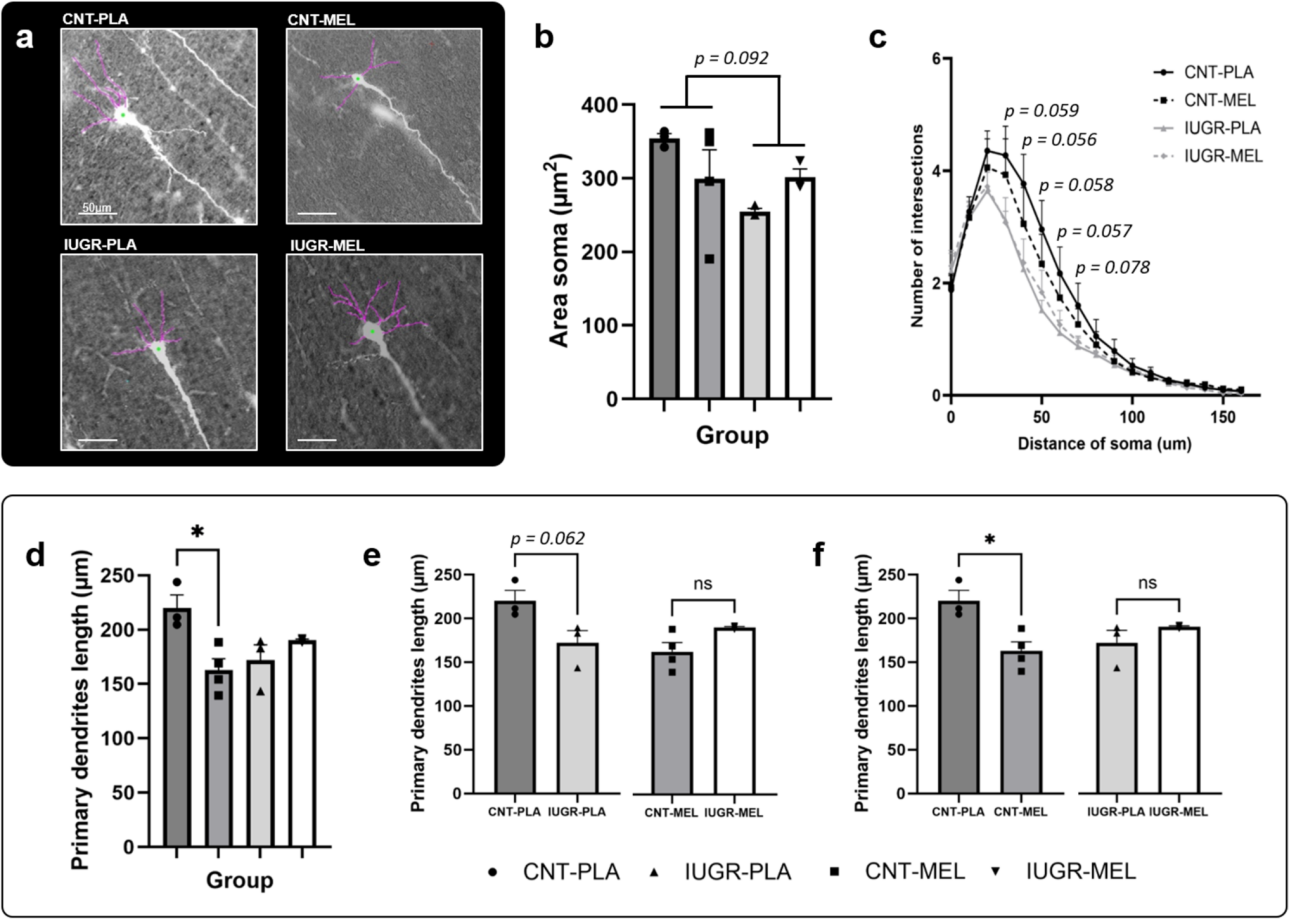
Neuronal arborization. Representative images of Sholl analysis with traced dendrites, scale bar = 50 µm (**a**). Area of the soma (**b**) and Sholl analysis (**c**) representing the number of intersections in relation of the distance to the soma (µm). Length of primary (**d**) dendrites depending on the treatment group. Pairwise comparisons depending on the model (**e**) and treatment (**f**) of primary dendrite length. An asterisk (*) represents a significant difference between groups at *p* < 0.05. Results are represented as mean ± S.E.M. Abbreviations: CNT, control; IUGR, Intrauterine growth restriction; PLA, placebo; MEL, melatonin

A two-way ANOVA (model x treatment) was used to study the endpoints related to neuronal arborization (Fig. 5a). A non-significant tendency of the model [F(1,9) = 3.547, *p* < 0.092] and the interaction between the model and treatment [F(1,9) = 3.821, *p* < 0.082] were observed regarding the area of the soma. The results suggest a decreased area of the soma in the IUGR group compared to CNTs, and a dual response to the MEL-treatment (Fig. 5b). Although not statistically significant, Sholl analysis presented a trend (*p* < 0.080) for the CNT groups to present a higher number of intersections compared to the IUGR groups (Fig. 5c).

Regarding primary dendrites length, a significant interaction between the model and treatment was observed [F(1,9) = 11.985, *p* = 0.007] (Fig. 5d). Further pairwise comparison showed a non-significant trend between CNT and IUGR treated with PLA [t = 2.563, d.f.4, *p* = 0.062], suggesting a potential decrease on primary dendrite length (Fig. 5e). Additionally, a significant difference was observed between CNT groups [t = 3.557, d.f.5, *p* = 0.016], showing a decrease in the MEL-treated group compared to PLA group (Fig. 5f). One-way ANOVA (group) showed significant differences in the length of primary dendrites between groups [F(3,9) = 5.450, *p* = 0.021]. Further *post-hoc* analysis found a decrease in the length of primary dendrites in the CNT-MEL compared to CNT-PLA (*p* = 0.017).

No significant differences between groups were observed in number of primary and secondary dendrites, nor the length of secondary dendrites (Supplementary Fig. S4).

### DAB Receptors

**Fig. 6:**
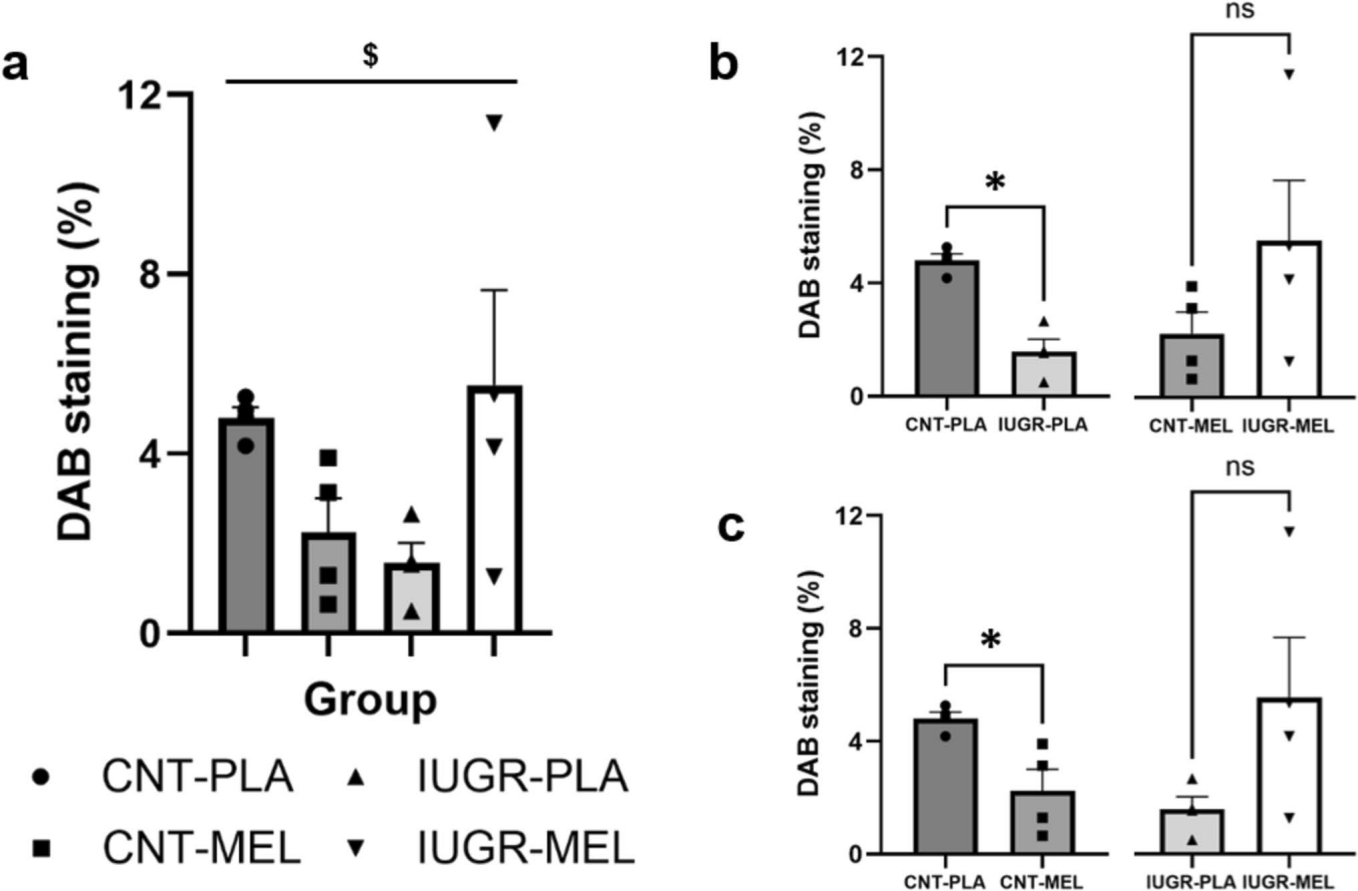
DAB staining of the MEL receptors. Percentage of DAB staining in the CA2 of the hippocampus depending on the treatment group (**a**). Pairwise comparisons based on the model (**b**) or the treatment (**c**). An asterisk (*) indicates a significant difference between groups, while the symbol ($) represents a significant interaction between the model and the treatment at *p* < 0.05. Results are represented as mean ± S.E.M. Abbreviations: CNT, control; IUGR, Intrauterine growth restriction; PLA, placebo; MEL, melatonin

The presence of MEL receptor 2 in the hippocampal CA2 region was assessed using DAB staining. The potential impact of MT2 on brain function was studied, considering the role of MEL receptors in mediating its effects. Differences in the percentage of DAB staining were evaluated through a two-way ANOVA (model x treatment). The analysis revealed a significant interaction between model and treatment [F(1,12) = 7.919, *p* = 0.016], as a dual response to MEL was observed depending on the model (Fig. 6a). Subsequent pairwise comparisons using t-tests showed significant differences between the CNT and IUGR groups treated with PLA [t = 6.485, d.f.6, *p* < 0.001], indicating a decrease in the percentage of MEL receptors in IUGR group (Fig. 6b). Additionally, although not statistically significant, an observable trend of MEL effects on the IUGR model was noted (n.s. *p* = 0.119), suggesting a potential increase in receptor percentages. Interestingly, significant differences depending on the treatment were observed in CNT group [t = 3.209, d.f.6, *p* = 0.008], indicating a reduction in MEL receptor percentages only within the CNT group treated with MEL (Fig. 6c).

## Discussion

The present study was designed to assess the potential neuroprotective effects of gestational MEL treatment on a rabbit model of surgically induced IUGR. Relevant neurodevelopmental endpoints, along with other basic parameters, were studied on the neonatal brains. Differences between CNT and IUGR were observed, consistent with previous reports in the literature. Our findings indicate that MEL treatment reversed some of these observed differences, indicating a neuroprotective role. Interestingly, a dual effect was noted depending if MEL was administered to CNT or IUGR fetuses, which requires further investigation. This study sheds light on the complex interplay between prenatal MEL exposure and neurodevelopment, highlighting pathways for future research and potential clinical applications.

### Perinatal, Placental and Neurodevelopmental Characteristics of the IUGR Model

As expected, significant differences were observed between CNT and IUGR groups across most of the studied endpoints. IUGR presented lower BW and a lower percentage of survival than CNT, which confirms the correct induction of the model, and aligns with the results previously obtained in our group [19]. However, no discernible effects were observed between groups in the functional evaluation. The test, adapted from Derrick et al. (2004) [25], was designed to assess severe motor and sensitive impairments secondary to severe and acute perinatal hypoxia–ischemia events in rabbit pups, and might not have been sensitive enough to detect more subtle functional effects at such young ages. Therefore, we suggest exploring alternative evaluation methods in future studies that would be able to assess other neurodevelopmental dimensions in rabbit pups that would be more appropriate for the model used in our research. Ischemia in the placenta emerged as a consistent histopathological difference among groups, although the specific regions affected varied between studies [19]. Regarding the analysis of brain endpoints, although both studies observed a non-significant decrease in the number of oligodendrocytes (*p* = 0.055), the main discrepancy was observed in neuronal arborization. More specifically, Pla et al. (2021) [19] observed an increased arborization in IUGR, which also correlated with subsequent in vitro studies [26], whereas in the present study we were not able to replicate this finding. One possible explanation is the difference in the microscopy techniques employed; our study did not utilize Z-stack imaging, potentially limiting our ability to discern differences at higher levels of arborization. Another important disparity between studies was the statistical analysis, with our study using the litter as the experimental unit. This decision was based on previous evidence, demonstrating that results obtained when using the litter as the statistical unit are comparable to those obtained when using individual pups, and more reliable from a biological relevance point of view, since it avoids the interpretation of strong adverse effects in one litter as general adverse effects in a whole study group, and therefore, it avoids overestimation of adverse effects [15]. Furthermore, this approach is consistent with the recommendations of the OECD guideline for developmental neurotoxicity, Test No. 426 [27].

### Effects of MEL on Placental Histology and Perinatal Survival

Gestational MEL treatment emerges as a promising therapeutic approach in reversing certain effects previously associated with IUGR. Firstly, MEL treatment notably decreased the percentage of calcifications observed in the decidua part of the placenta within both the CNT and IUGR groups. A higher presence of calcifications in the placenta during the final stages of pregnancy is commonly observed in humans, particularly in IUGR pregnancies [28]. To the best of our knowledge, this is the first evidence of MEL reducing the percentage of calcifications in the placenta in both CNT and IUGR groups. Nonetheless, the well-known antioxidant properties of MEL had already been associated with general improvements in placental health. These include enhancing nutrient transfer and vascular dynamics [29], reducing mitochondrial damage [30], and modulating immune responses by decreasing the levels of pro-inflammatory cytokines [31].

Regarding perinatal results, MEL treatment did not reverse the lower BW and reduced survival rate in the IUGR group. Additionally, MEL-treated animals exhibited significantly lower birth weights compared to the CNT group. We hypothesize that this observation may be attributed to MEL treatment potentially enhancing survival rates at lower birth weights within the IUGR cohort, as indicated by the lower BW observed in the MEL-treated groups. This observation is consistent with the literature. Two studies using sheep IUGR models found that MEL not only failed to increase BW [32], but also induced a decrease in offspring BW [14]. Furthermore, an investigation involving two murine models of fetal growth restriction (FGR) found that MEL increased fetal weight in wild-type mice but not in the FGR group [33].

### Effects of MEL on Neurodevelopment

MEL treatment reversed the impact of IUGR in brain development by significantly increasing the number of oligodendrocytes, rendering it comparable to that of the CNT group. These results are in line with Kühne et al. (2022) [4], who also observed that MEL increased the number of O4 + cells in IUGR in an in vitro neurosphere culture. In fact, MEL promoted oligodendrocyte differentiation, which was delayed in the IUGR group, resulting in an increased number of O4 + positive cells [4]. In a study in ewes, the authors found no differences in oligodendrocyte lineage (Olig2 +) cell counts in the brain, but they did find differences in the levels of the myelin protein CNPase between groups [32]. In particular, they observed a decrease in CNPase fiber density in fetal growth-restricted individuals, which was reversed in MEL-treated groups; with total levels depending on the cerebral region studied. Similar results regarding CNPase levels were previously reported by Miller et al. (2014) [13]. Given that myelin is produced by mature oligodendrocytes, the authors hypothesized that MEL treatment may be promoting normal maturation events [32]. In light of these studies, we believe that the observed effects in the MEL-treated group are due to MEL’s role in this maturation process of oligodendrocytes.

Regarding neuronal arborization, our results showing a reduction in soma area and primary dendrites length in IUGR compared to CNT group are consistent with those found by García-Chávez et al. (2008) [34] in the same brain region following global cerebral ischemia in adult rats. However, the authors found that these alterations were prevented in animals that received MEL [34]. Another study discovered that MEL stimulated dendrite maturation in the brain of male mice, increasing total length and complexity in the hippocampus, an effect also observed in vitro with primary cultured neurons [35]. However, it is important to note that both studies used adult animals, with MEL administration by intravenous infusion for 6 h [34] and by intraperitoneal injection for 15 days [35], while our administration was oral to the mother for 6 consecutive days during gestation. Evidence towards maturation and neurogenesis in the hippocampus after chronic exposure to MEL [11] allows us to speculate that our treatment period was not sufficient to observe significant amelioration. Nonetheless, our lack of beneficial effects of MEL on neuronal arborization aligns with previous research conducted by our group: Kühne et al. (2023) [26] also did not observe positive effects on the neurosphere model after in vivo MEL treatment. Moreover, our determinations were performed on the neurons in layer II and III of the frontal cortex, which, compared to the hippocampus, present a lower presence of MEL receptor 2, primarily found in deeper layers [36].

Based on previous results, it is important to note that MEL's positive effects on neurodevelopment are not due to an increase in body weight. Instead, these effects likely result from a direct action of MEL on the nervous system, the placenta, or a combination of both.

### Effects of MEL on its Receptors

MEL has been described to produce its effects both dependently and independently of its receptors [36, 37]. For this reason, we investigated changes in the percentage of MT2 to understand its potential role in brain alterations. We observed a decrease in the percentage of MT2 receptors in IUGR compared to CNT, with a tendency for this reduction to be reversed by MEL treatment. MT2 receptors have been previously related, both in vitro and in vivo, with neurogenesis and axon formation in the hippocampus [38]. The decrease observed in IUGR group is consistent with a previous study by Berbets et al. (2021) [39], who found that the expression of MEL 1 A and 1B receptors (corresponding to MT1 and MT2, respectively) was decreased in the placenta of women with FGR. In contrast, a study with twin-bearing ewes subjected to FGR induction found no significant differences on MEL receptor 1B expression in the offspring brains [32]. However, when they assessed receptor density, they did find an increase in periventricular white matter (PVWM) in the FGR group, which was reverted by MEL administration, leading to a decreased density compared to the untreated animals [32]. These results contrast with those found in the present study in IUGR and with MEL treatment. While not statistically significant, we found a trend towards an increase of MEL receptors in the treated group. These discrepancies may depend on the specific brain region studied; we focused on the CA2 in the hippocampus, whereas Malhotra et al. (2024) [32] focused in the PVWM. Different distributions depending on the brain region have been previously reported in the literature [36].

### Dual Effect of MEL

Finally yet importantly, we observed a dual effect of MEL treatment depending on the model on several endpoints. An improvement was noted in the IUGR group, while a detrimental effect was observed in the CNT group, particularly in parameters such as the number of oligodendrocytes, primary dendrite length, and the presence of MT2 in the hippocampus. This dual effect deserves further attention, as it may have important implications for the safety profile of MEL administration to healthy patients. Additionally, it sheds more light on the ongoing debate regarding the safety of gestational MEL administration. Currently, there is no consensus on this matter, with some studies reporting negative effects of MEL [14], while others have found it to be safe [13, 40]. This is especially relevant considering that MEL is available over-the-counter in many countries. González-Candia et al. (2016) [14] observed adverse effects of antenatal MEL treatment in an IUGR sheep model of chronic-hypoxia due to high altitude. The authors observed a greater restriction of growth in the MEL-treated group compared to the CNT group. Additionally, other studies found a link between gestational MEL treatment and reproductive impairments later in life, such as altered levels of sexual hormones like prolactin [41], delayed vaginal opening [42] and altered testicular function [43]. Interestingly, previous in vitro studies with neurospheres observed that exposure to 1 µM MEL increased the percentage of oligodendrocytes in IUGR neurospheres, while 10 µM MEL reduced viability to below 70% in CNT ones [4].

### Limitations and Strengths

A potential limitation of the study is that the sex of the pups could not be assessed within the study groups, as it was not clearly identifiable at the neonatal stage. However, a study by van der Merwe [44] used SRY-gene detection by PCR to identify the male rabbit pups and found no overall association between sex and the studied outcomes. Another limitation is the absence of placental weight data. Previous findings in the same animal model have shown that surgical induction of placental insufficiency leads to a significant reduction in placental weight. Notably, data published by Pla et al. 2020 [19] reported that although placental weight was lower in the IUGR group compared to controls, the difference did not reach statistical significance. Additionally, potential limitations of MEL as a prenatal therapy for treating IUGR are its short half-life in adults of approximately 45 min and low oral bioavailability, which typically ranges from 3 to 33% due to first-pass metabolism in the liver [45]. To overcome these limitations, some studies have employed strategies such as intravenous continuous infusion [13, 32] or administration through drinking water [33]. To address these concerns, our study design closely resembles the expected clinical route of administration, improving the translational potential and applicability of our findings to potential clinical settings. Another strength of the study is the inclusion of a CNT group, which allows for the assessment of MEL's effects in healthy patients. Unlike most studies that focus solely on the IUGR group, our study provides crucial safety information, helping us identify and address the dual effect of MEL.

## Conclusions

Our findings provide valuable insights into the neuroprotective effects of gestational MEL administration in an in vivo IUGR rabbit model. The most significant improvements in IUGR were a reduction in placental calcifications and an increase in oligodendrocyte count, indicating improved placenta preservation and enhanced oligodendrocyte maturation. MEL receptors appear to play a crucial role in mediating these beneficial effects in IUGR subjects. However, our study indicates a dual effect of MEL, dependent on the baseline condition. While MEL shows promise as a gestational treatment for confirmed IUGR cases, caution and strict monitoring are advised in healthy pregnancies. Overall, these results raise several intriguing questions about the neurodevelopmental impact of MEL in healthy individuals. Given the potential implications for human health, further research is required.

## Supplementary Information

Below is the link to the electronic supplementary material.

Supplementary file1 (DOCX 771 KB)

## Data Availability

The data that support the findings of this study are available from the corresponding author upon reasonable request.
