# Supplementary material for: Dual Effect of Melatonin in Fetal Brain: Structural and Cellular Implications in a Rabbit Model of Intrauterine Growth Restriction
Source: Mol Neurobiol. 2025 Aug 1;62(12):16119–32. doi: 10.1007/s12035-025-05032-y (PMC12559042; doi:10.1007/s12035-025-05032-y)
Supplement: Supplementary file 1 — Supplementary file1 (DOCX 771 KB) [file 12035_2025_5032_MOESM1_ESM.docx]

**Supplementary material**

Neuroprotective potential of gestational melatonin: insights from a rabbit model of intrauterine growth restriction.

Laia Guardia-Escote^1,2,*^, Yvan Gómez^3,*^, Mercè Fuentes-Amell^1^, Carlota Rovira^4^, Eduard Gratacós^3,6^, Britta Anna Kühne^1,3,5^, Marta Barenys^1,5^, Miriam Illa^3,7, 🖂^ .

^*^ *Both authors contributed equally to this manuscript*

^1^ GRET and Toxicology Unit, Department of Pharmacology, Toxicology and Therapeutical Chemistry, Faculty of Pharmacy, University of Barcelona, Barcelona (Spain). [laia.guardia@ub.edu](mailto:laia.guardia@ub.edu) (LG-E); [merce.fuam@gmail.com](mailto:merce.fuam@gmail.com) (MF-A); [britta.kuehne@bfr.bund.de](mailto:britta.kuehne@bfr.bund.de) (BAK); [mbarenys@ub.edu](mailto:mbarenys@ub.edu) (MB).

^2^ Department of Psychology, Faculty of Psychology, Universitat Rovira i Virgili, Tarragona (Spain).

^3^ BCNatal | Fetal Medicine Research Center, Hospital Clínic i Hospital Sant Joan de Déu, Universitat de Barcelona, Barcelona (Spain). [yvan.gomez1@gmail.com](mailto:yvan.gomez1@gmail.com) (YG); [gratacos@clinic.cat](mailto:gratacos@clinic.cat) (EG); [miriam.illa@sjd.es](mailto:miriam.illa@sjd.es) (MI).

^4^ Department of Anatomical Pathology, Hospital Sant Joan de Déu, Barcelona (Spain). [carlota.rovira@sjd.es](mailto:carlota.rovira@sjd.es) (CR).

^5^ German Centre for the Protection of Laboratory Animals (Bf3R), German Federal Institute for Risk Assessment (BfR), Berlin (Germany).

^6^ Institut d'Investigacions Biomèdiques August Pi i Sunyer (IDIBAPS), Barcelona and Centre for Biomedical Research on Rare Diseases (CIBERER), Barcelona (Spain)

^7^ Primary Care Interventions to Prevent Maternal and Child Chronic Diseases of Perinatal and Developmental Origin Network (RICORS), RD21/0012/0003, Instituto de Salud Carlos III, Madrid, Spain

🖂*Corresponding author*: [miriam.illa@sjd.es](mailto:miriam.illa@sjd.es); BCNatal | Fetal Medicine Research Center, Hospital Clínic i Hospital Sant Joan de Déu, Universitat de Barcelona, and Institut de Recerca Sant Joan de Déu (IRSJD), Fundació Sant Joan de Déu, Barcelona (Spain). ORCID: 0000-0002-8366-830X

**Supplementary table 1.** Functional evaluation at PND 1.

| IUGR RABBIT MODEL | | | | | | | | | |
| --- | --- | --- | --- | --- | --- | --- | --- | --- | --- |
| **Fetus ID: \|**______________\| | | | | **Chip code:** \|_\|_\|_\|_\|_\| | | | | | **Postnatal day:** \|_\|_\| |
| **Date of evaluation:** \|_\|_\| \|_\|_\| \|_\|_\| | | | | **Classification**: \|_\| (0-CNT, 1-IUGR) | | | | | **Treatment:** \|_\| (0-PLA, 1-MEL) |
| **NEUROLOGICAL EVALUATION** *(0: worst – 3/4: best)*  **Weigh fetus** **at +1P:** \|_\|_\|,g | | | | | | | | | |
| A- Posture | | | | | | | **F- Intensity** | | |
| **0** | Lays supine | | | | | | **0** | No movement | |
| **1** | Lays on side | | | | | | **1** | Slight activity | |
| **2** | Cannot maintain prone position | | | | | | **2** | Distinct forceful movements | |
| **3** | Prone position | | | | | | **3** | Rapid forceful movements | |
| **B- Righting reflex (10 tries)** | | | | | | | **G- Duration (observation for 1 minute)** | | |
| Nº times turns prone from supine \|__\| | | | | | | | **0** | No movement | |
| C- Tone | | | | | | | **1** | Activity <20 sec | |
| **0** | Limb rigid in flexion or extension | | | | | | **2** | Activity 20-40 sec | |
| **1** | Increase in tone, passive movement difficult | | | | | | **3** | Activity >40 sec | |
| **2** | Marked increase in tone but limb is easily flexed | | | | | | **H- Lineal movement ( 1 min, distance 15 cm)** | | |
| **3** | Slight increase in tone when limb is moved | | | | | | Nº of times crosses perpendicular line \|__\| | | |
| **4** | No increase in tone | | | | | | I- Shortest fore-hindpaw distance | | |
| D- Circular motion (one minute) | | | | | | | 5 measurements (if pup walks in straight line) \|__\| | | |
| **0** | No movement | | | | | | J- Sucking and swallowing | | |
| **1** | Slight movement, slight jump | | | | | | **0** | No movement of jaw, milks dribbles out completely | |
| **2** | Good range of motion, maintains for 1-2 steps, occasional jump | | | | | | **1** | Some movement of jaw and neck, most of milk dribbles out | |
| **3** | Entire range of motion, at least 3 steps, rapid jumps | | | | | | **2** | Definite suck and swallow, some milk in nose | |
| E- Locomotion: | | H | | | **FL** | HL | **3** | Good suck and swallow, no milk in nose | |
| **0** | No movement \|__\| \|__\| \|__\| | | | | | | K- Head turning during feeding | | |
| **1** | Slight movement \|__\| \|__\| \|__\| | | | | | | **0** | No movement | |
| **2** | Distinct movement \|__\| \|__\| \|__\| | | | | | | **1** | Slight occasional movement of head | |
| **3** | Rapid movement \|__\| \|__\| \|__\| | | | | | | **2** | Distinct movement of head | |
|  | | | | | | | **3** | Rapid forceful movements of head and body | |
| **ODOUR TEST** | | | Aversive response \|_____\| (0-No,1-subtil, 2-low response, 3-correct)  Latency in aversive response \|___\| (seconds) | | | | | | |

Abbreviations: PND, postnatal day; CNT, control; IUGR, Intrauterine growth restriction; PLA, placebo; MEL, melatonin; BW, body weight.

**Supplementary table 2.** Histopathological assessment of placenta.

| Endpoints | Score | Description |
| --- | --- | --- |
| Ischemia phase 1 | % of affected area | Light pink staining, nuclei are still visible |
| Ischemia phase 2 | % of affected area | Light pink staining, nuclei show disintegration and appear as dark nuclear dust |
| Polymorphonuclear cells | Presence (Y/N) | Indicates whether polymorphonuclear (PMN) cells are observed in the tissue |
| Fibrosis | % of fibrotic tissue | Cloudy light pink structures lacking nuclei, indicating fibrotic tissue formation. |
| Fibrin | % of tissue with fibrin presence | Cloudy light pink structures with a less dense, network-like appearance, suggesting fibrin deposition. |
| Calcifications | % of tissue with calcifications | Dark purple staining indicating the most advanced stage of cell necrosis, where calcifications are present. |
| Vascular collapse | 0 = Unremarkable, 1 = Minimal, 2 = Mild, 3 = Moderate, 4 = Marked, 5 = Severe | Collapse of the stromal structure in the labyrinth; a reduction in the width of blood vessels and tissue. |

**Supplementary table 3.** Number of pups per litter.

| PLA *Mean number of pups per litter: 6.00 ± 1.83* | MEL *Mean number of pups per litter: 6.38 ± 2.62* |
| --- | --- |
| n = 8 (2 CNT + 6 IUGR) | n = 7 (1 CNT + 6 IUGR) |
| n = 3 (2 CNT + 1 IUGR) | n = 7 (2 CNT + 5 IUGR) |
| n = 7 (4 CNT + 3 IUGR) | n = 4 (2 CNT + 2 IUGR) |
| n = 4 (4 CNT + 0 IUGR) | n = 3 (3 CNT + 0 IUGR) |
| n = 7 (3 CNT + 4 IUGR) | n = 8 (3 CNT + 5 IUGR) |
| n = 6 (3 CNT + 3 IUGR) | n = 11 (3 CNT + 8 IUGR) |
| n = 7 (3 CNT + 4 IUGR) | n = 7 (2 CNT + 5 IUGR) |
|  | n = 4 (2 CNT + 2 IUGR) |


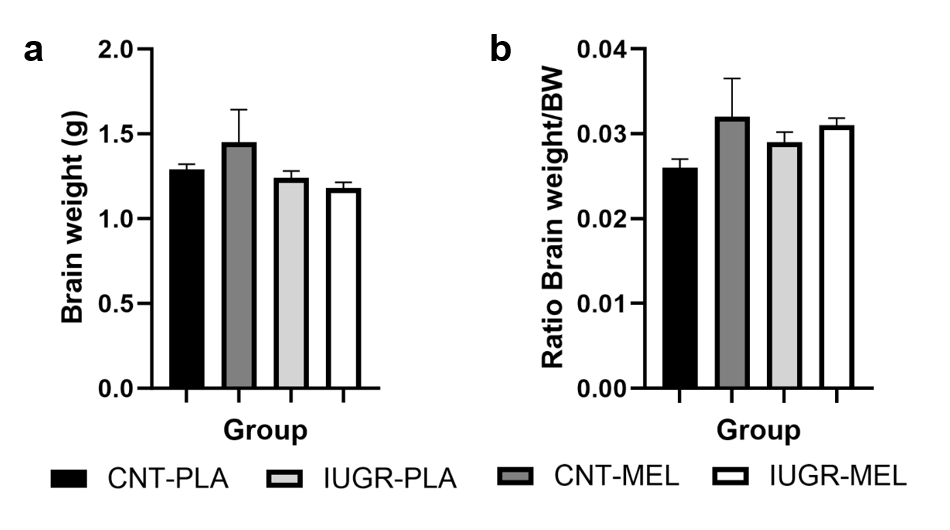


**Figure S1. Perinatal data.** Brain weight (a) and the ratio between brain weight/BW (b). Abbreviations: CNT, control; IUGR, Intrauterine growth restriction; PLA, placebo; MEL, melatonin; BW, body weight.


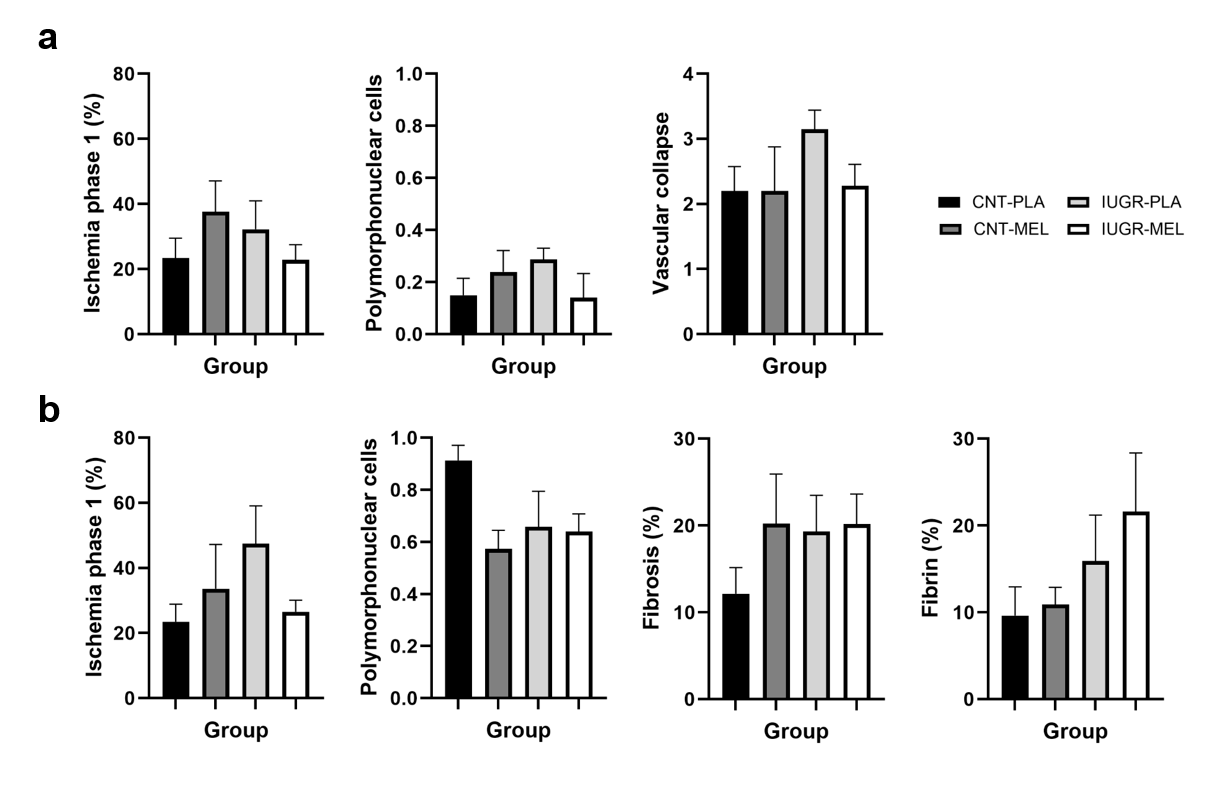


**Figure S2. Placenta histopathology.** Histopathological endpoints analyzed in the labyrinth (a) and the decidua (b) part of the placenta. Abbreviations: CNT, control; IUGR, Intrauterine growth restriction; PLA, placebo; MEL, melatonin.


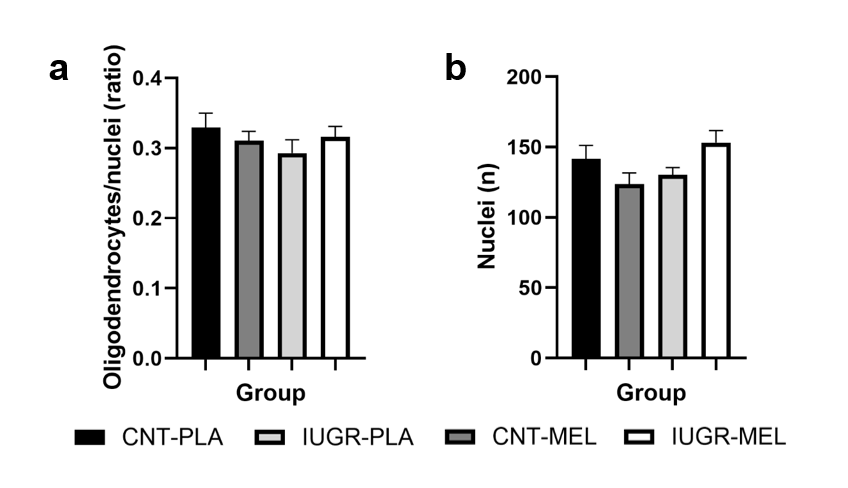


**Figure S3. Oligodendrocyte study.** Number of oligodendrocytes/number of total cells (a) and number of nuclei (b). Abbreviations: CNT, control; IUGR, Intrauterine growth restriction; PLA, placebo; MEL, melatonin.


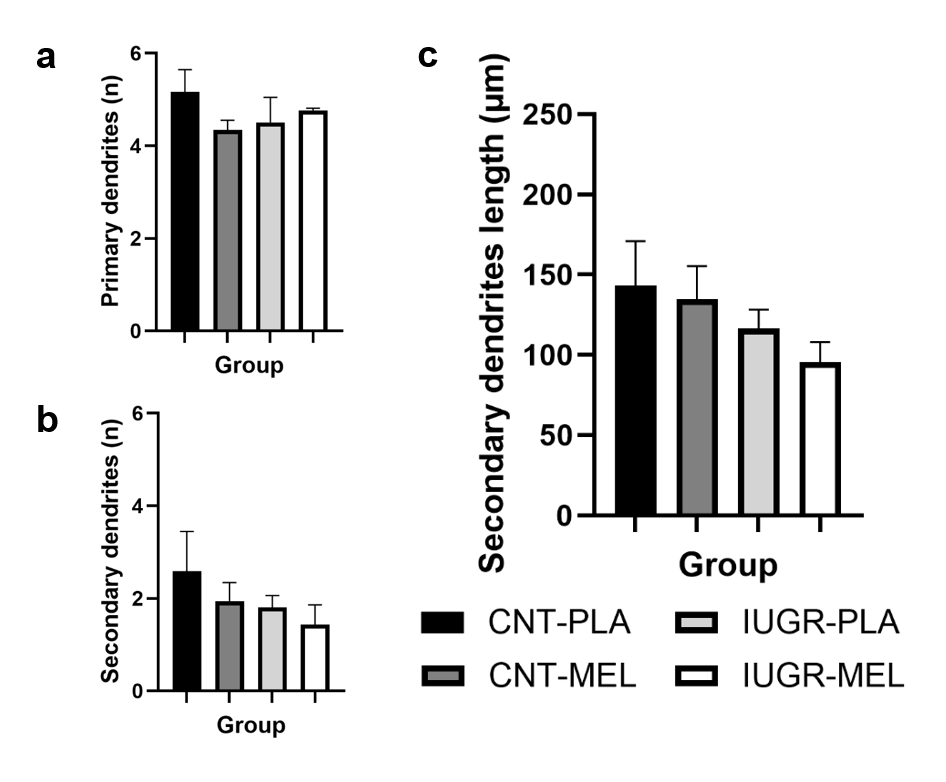


**Figure S4. Neuronal arborization.** Number of primary (a) and secondary (b) dendrites, and secondary dendrites length (c). Abbreviations: CNT, control; IUGR, Intrauterine growth restriction; PLA, placebo; MEL, melatonin.
